# Supplementary material for: Assessing the pattern of key factors on women’s empowerment in Bangladesh: Evidence from Bangladesh Demographic and Health Survey, 2007 to 2017–18
Source: PLoS One. 2024 Mar 29;19(3):e0301501. doi: 10.1371/journal.pone.0301501 (PMC10980244; doi:10.1371/journal.pone.0301501)
Supplement: Attachment — Submitted filename: Response to reviewers.docx [file pone.0301501.s001.docx]

**Authors’ responses to the points raised by the Academic Editor and reviewers**

First of all, we would like to thank the Academic Editor and the reviewers for reviewing our joint work entitled “Assessing the pattern of key factors on women’s empowerment in Bangladesh: evidence from Bangladesh Demographic and Health Survey, 2007 to 2017-18” and providing his valuable suggestions about our manuscript. All the suggestions raised by the Academic Editor and reviewers have been addressed in the revised version which are categorically pointed out in the followings:

1. We are suggested to re-write the methods, results, and conclusion in the ‘Abstract’ section, which is done accordingly.
2. According to the valuable suggestions, ‘Introduction’ is shortened and given appropriate citations.
3. We are asked not to use table for data description, which has been removed from the revised manuscript.
4. We are advised to explain more about PCA, to mention how many variables remained after PCA, about the methodology of doing it, to provide the way we have categorized the obtained score on what basis. It is added in the respective section ‘Outcome variable’.
5. We are suggested to add citations in Table 1, which is done accordingly.
6. In ‘Statistical Analyses’ section, a specified level of significance and a technique of assessing goodness of fit of the fitted model are mentioned. Explanation about the statistical software with their uses are given.
7. ‘Results’ section has been re-written with proper suggested tables and interpretations.
8. ‘Discussion’ section has been re-written according to the suggestions.
9. ‘Conclusion’ section has been shortened and re-modified accordingly.
